# Disaster response, confined space medicine and safety systems against railway accidents: 2023 Odisha railway accident from India

**DOI:** 10.1002/puh2.135

**Published:** 2023-11-10

**Authors:** Aravind P. Gandhi, Bijaya Kumar Padhi

**Affiliations:** ^1^ Department of Community Medicine All India Institute of Medical Sciences Nagpur India; ^2^ Department of Community Medicine and School of Public Health Postgraduate Institute of Medical Education and Research Chandigarh India

**Keywords:** confined space medicine, disaster management, railway accident

## Abstract

Railways are a major mode of transportation for passengers as well as goods. Across the world, minor and major accidents involving railways causing damage to life and property have been reported. The recent triple train derailment and collision near a small railway station in the state of Odisha, India, on 02.06.2023 is a major calamity. The accident resulted in 292 deaths, and more than 1000 injuries were attributed to the accident. In its comprehensive enquiry report, the Commissioner of Railway Safety found lapses in the signalling mechanism and oversight as the reasons for the accident. Immediate rescue, emergency transport and treatment are essential in reducing the mortality and morbidity of such accidents. The relatively newer domain under pre‐hospital and disaster medicine is confined space medicine, which deals with patients trapped in places with limited access to conventional intervention and reduced ventilation. Confined space medicine employs a multi‐hazard and interprofessional approach to treating individuals who are trapped in a structural collapse. It is also essential to critically evaluate and disseminate the entire disaster response systems that functioned during and after the railway accident to identify the existing system's best practices, bottlenecks, and gaps. The train's speed, lack of track and signal maintenance, absence of safety oversight, driver fatigue and distractions are some major determinants of train accidents, highlighting the need for comprehensive planning and training in rail safety. It is essential to improve the timely implementation of maintenance activities and completion of accident inquiries on time. A comprehensive review of the preparedness, rescue and response must be undertaken at the earliest possible opportunity to prevent and prepare for any such disasters in the future.

## INTRODUCTION

Railways are a major mode of transportation for passengers as well as goods. Ecologically, it has been one of the mass transport modalities with the least carbon emissions. India is one of the countries with the largest railway network and services providing services that crisscross the length and breadth of the sub‐continent. Indian Railway (IR) commutes around 22.2 million passengers daily through its network of 21,648 trains. Accidents involving railways are not uncommon [1]. Across the world, minor and major accidents involving railways causing damage to life and property have been reported. Railway accidents can happen through various modes such as collisions, derailment, fire, level crossing and miscellaneous. Regarding mechanism, ‘jack‐knifing’ and ‘overriding’ are the two most common phenomena behind railway accidents. Jack‐knifing is when the carriages go off the tracks when the train hits and crashes into each other. This causes the sides of the carriages to buckle inwards, harming the passengers inside. Overriding occurs when one carriage climbs over another, landing on and crushing the passengers beneath it [2]. In 2021, 17,993 cases of railway accidents were responsible for 16,431 fatalities and 1852 injuries in India [3]. However, single events of mass casualties involving fatalities in hundreds and injuries in thousands of passengers are not common. The recent triple train derailment and collision near a small railway station in Odisha, India, is one such major calamity.

### Odisha train accident–sequence of events

The Odisha railway accident happened at around 7 pm Indian Standard Time (IST) on 02.06.2023 at Bahanaga Bazar railway station, which is 170 km from Bhubaneshwar, the capital city of Odisha. The accident resulted in 292 deaths, and more than 1000 injuries were attributed to the accident [4]. Initial reports suggested the following potential sequence of events: collision between an immobile freight train parked at the loop line of the Bahanaga Bazar railway station and a moving passenger train inadvertently entering the same loop line, leading to the derailment of the passenger train compartments. The derailed compartments hit the tail end of the third train, which was another passenger train running on a parallel main line simultaneously, causing the derailment of the third train [5]. The responsible authority, the Commissioner of Railway Safety, in its comprehensive enquiry report, found lapses in the signalling mechanism and oversight as the reasons for the accident [6].

### Pre‐hospital and disaster medicine

Immediate rescue, emergency transport and treatment are essential in reducing the mortality and morbidity during such accidents. Pre‐hospital and disaster medicine plays a vital role during the early rescue and restoration operations following the train accident. Although early extrication is the best chance for survival, train crashes can trap survivors with a time‐consuming physical rescue process. In such a scenario, ensuring the sustenance of the trapped survivors' lives in the tight space is essential [7]. The relatively newer domain under pre‐hospital and disaster medicine is confined space medicine, which deals with patients trapped in places with limited access to conventional intervention and reduced ventilation. When basic medical procedures become challenging, confined‐space medical techniques are essential. These techniques are highly effective in preventing the occurrence of crush syndrome, which can result from prolonged entrapment [7].

### Confined space medicine

Confined space medicine employs a multi‐hazard and interprofessional approach to treating individuals who are trapped in a structural collapse. This specialised field also faces distinctive challenges regarding the initial management of airways in such victims, as well as the treatment of crush syndrome [8]. Adoption of confined space medicine during such disasters can reduce mortality since timely interventions can be ensured during the ‘golden hour’ following the incident. It will prevent secondary injuries and complications from the accident, such as crush syndrome and, in turn, mortality.

### Rescue and response during disasters

Critical evaluation and dissemination of the entire disaster response systems that functioned during and after the railway accident will enable better preparedness and response in the future. Nagata et al. presented an extensive and critical review of the “Express Railway Disaster in Amagasaki” in Japan, 2005 [7]. Early response and volunteers ensured that no life was lost due to emergency transport delays and incorrect triage. They flagged critical issues in the communication systems and chain of command at the crash site, with a recommendation for an incident command system for better disaster coordination. India adopted the Incident Response System (IRS) under its disaster management policy [9].

### Disaster response and medical relief in the Odisha train accident

Following the Odisha railway accident, the IRS was activated. Through the District Disaster Management Authority under the District Magistrate, the Special Relief Commissioner deployed the various components of the IRS to enable seamless and coordinated disaster management. The IR, the fire and rescue department of Odisha state were rushed to the spot and commenced the rescue of passengers trapped within and under the derailed compartments. Considering the quantum of the accident, emergency medical transports were mobilised from the neighbouring districts. The pre‐hospital emergency medical services in the area where the accident occurred have Geographical Information System (GIS) and Geographical Positioning System (GPS) enabled free‐of‐cost public ambulances. Two levels of ambulance – Advance Life Saving (ALS) and Basic Life Support (BLS) – are available with appropriate equipment to manage the pre‐hospital care. Each ambulance is crewed by adequately qualified and trained emergency medical technicians capable of providing pre‐hospitalisation care at the event spot and during transit to the healthcare facility [10]. Initially, the injured passengers were taken to the Balasore district hospital and government Soro hospital (within the Balasore district), which are secondary care centres. The primary health and community health centres near the accident site were overwhelmed with patients. The hospitals were working well beyond their operational and technical capacity, a major challenge. In order to overcome the complexity of the injuries in terms of quantity and criticality, injured passengers were referred to SCB Medical College Hospital in Cuttack, a tertiary care institute. Doctors from the apex healthcare institute of India – All India Institute of Medical Sciences (AIIMS) – were flown in to manage the critical cases [11]. One of the challenges during the management was language. Most of the injured spoke only non‐Oriya languages (Oriya is the local language in Odisha); hence, communication was difficult. This might be a persistent issue in railway accidents involving long‐distance trains in a linguistically diverse country like India, which needs to be factored in while planning pre‐hospital and hospital care.

### Determinants of train accidents

The speed of the train, lack of track and signal maintenance, and the absence of safety oversight are some of the major determinants of train accidents. The lack of attention from train drivers due to fatigue or distractions diminishes their driving capability, which is the primary human‐related cause of train mishaps [12]. Planning in terms of risk assessment, infrastructure maintenance, emergency preparedness and regulatory compliance ensures rail safety. Providing basic safety training to all railway employees, specialised training for drivers with refresher courses, mock drills, training on new technologies and strategies to tackle human factors such as fatigue forms an integral part of rail safety. With India introducing and envisaging to launch semi‐high speed and high‐speed railways systems across the country, the organisation must focus on the safety systems adequately and appropriately, more than ever.

### Challenges identified and the way forward in railway disasters

One of the major challenges during such a large disaster is the lack of a mechanism to swiftly arrange for adequate healthcare facilities to treat the injured, which led to overcrowding of the existing facilities. It would be prudent to have contingency plans and pre‐identified facilities (within and nearby districts) by the respective district disaster management authority as per the quantum of victims for efficient and timely management of large disasters. Due to the disaster's nature, the identification of the dead passengers was another challenge. Primary identification methods such as fingerprinting, forensic odontology and DNA analysis can be utilised to verify the dead. Strengthening the existing IRS for disaster response by taking the Odisha train accident as a case study can be done. Continuous feedback during the specific incident and after the incident helps in improving the disaster response rate and capability. India has started deploying the indigenously developed low‐cost collision avoidance system named ‘Kavach’. As the head‐on collision was between a passenger and a freight train, including and testing, the freight trains under the Kavach are vital. Moreover, as highlighted by the Comptroller and Auditor General of India, it is essential to improve the timely implementation of maintenance activities and the completion of accident inquiries on time [1]. It is also recommended to undertake a comprehensive Hazard, Vulnerability and Risk (HVR) of the railway system.

## CONCLUSION

Overall, railway accidents are preventable with adequate monitoring and prompt adherence to the safety systems. The entire railway network must be installed with novel anti‐collision systems, such as Kavach, to add a better layer of protection. A comprehensive review of the accident and its events must be undertaken. This includes the root‐cause analysis of the accident and the further evaluation of the effects of the rescue and relief efforts. Confined‐space medicine has a high scope in pre‐hospital medicine during disasters. It needs to be introduced into the disaster medicine curriculum, and specialists can be trained at district‐level hospitals in India.

## AUTHOR CONTRIBUTIONS

*Conceptualisation; methodology; investigation; formal analysis; writing – original draft; writing – review and editing*: Aravind P Gandhi. *Conceptualisation; methodology; data curation; validation; supervision; project administration; writing – review and editing*: Bijaya Kumar Padhi.

## CONFLICT OF INTEREST STATEMENT

The authors declare no conflicts of interest.

## Data Availability

The original contributions presented in the study are included in the article/Supporting Information; further inquiries can be directed to the corresponding author(s).
